# Time to Hypothyroidism Following Hemithyroidectomy

**DOI:** 10.7759/cureus.32837

**Published:** 2022-12-22

**Authors:** Christa R Abraham, Ashar Ata, Steven C Stain, Zeinab M Khalaf, Yusef Hazimeh

**Affiliations:** 1 Surgery, Albany Medical College, Albany, USA; 2 Surgery, Lahey Hospital and Medical Center Burlington, Lahey, USA; 3 Endocrinology, Diabetes and Metabolism, Faculty of Medical Sciences, Lebanese University, Beirut, LBN; 4 Endocrinology, Faculty of Medical Sciences, Lebanese University, Beirut, LBN

**Keywords:** thyroid stimulating hormone tsh, body mass index: bmi, euthyroid, hemi-thyroidectomy, hypothyroidism

## Abstract

Background

The time to hypothyroidism post hemithyroidectomy is variable. There are multiple risk factors for developing hypothyroidism. The aim of this study was to identify the time of hypothyroidism and other predictors of hypothyroidism in euthyroid patients following hemithyroidectomy.

Methods

This was a retrospective study. Of 170 euthyroid patients who underwent hemithyroidectomy for benign disease between 2006 - 2014, age, gender, pre-operative thyroid function tests, body mass index (BMI), and other co-morbidities were examined to determine predictors of early (<3 months) or late (>3 months) hypothyroidism. A high normal preoperative thyroid stimulating hormone (HN-TSH) was defined as ≥2.01 uIU/ml, and a low normal TSH (LN-TSH) was defined as <2.01 uIU/ml.

Results

A total of 63 of the 170 patients (37%) became hypothyroid. At 3 months, 21.5% of patients were hypothyroid. At 6 months after operation, an additional 5% had become hypothyroid, and after 1 year, 8% more were hypothyroid. The only independent predictor of hypothyroidism was preoperative HN-TSH (≥2.01) (p<0.001) on multivariate analysis.

Conclusion

In addition to known predictors of hypothyroidism following hemithyroidectomy for benign disease, such as the size of the thyroid remnant, a history of neck irradiation, and coexisting thyroid autoimmune disease, a BMI ≥35 kg/m^2^, age ≥45, and preoperative HN-TSH are risk factors for postoperative hypothyroidism within 3 months of operation. Such patients should be closely monitored.

## Introduction

Prior studies have demonstrated that the incidence of hypothyroidism after hemithyroidectomy ranges from 10% to 42.6% [1,2,3]. Previously identified risk factors for hypothyroidism include normal but sub-optimal pre-operative thyroid function based on high normal thyroid stimulating hormone (HN-TSH), low free thyroxine-4 levels (Free T4), lymphocytic thyroiditis on histology or serology, and an inadequate residual thyroid remnant [1,4,5]. Predicting which patients will develop new onset hypothyroidism after hemithyroidectomy and the time to hypothyroidism remains a challenge for surgeons.

The goal of this study was to identify the non-modifiable risk factors associated with post-operative hypothyroidism including age, weight, gender, and pre-operative thyroid function, to determine whether early (<3 months) or late onset hypothyroidism could be predicted.

## Materials and methods

Study design

This is a retrospective study aiming at determining predictors of postoperative hypothyroidism. Data related to all patients operated by hemithyroidectomy (by a single surgeon), and admitted to our institution between August 2006 and July 2014, were viewed. The inclusion criterion was euthyroid patients older than 18 years. The exclusion criteria were age less than 18 years, thyroid cancer patients, and total thyroidectomy. Hemithyroidectomy was defined as the resection of one thyroid lobe with isthmusectomy. The indications for operation were diagnostic intent based on preoperative cytology or therapeutic intent for compressive symptoms.

A total of 177 patients met the inclusion criteria. These patients were followed for 8 years.

Variables and measurement

The clinical variables evaluated included age, gender, co-morbidities, thyroid function, and obesity.

Thyroiditis was based on either preoperative serological evidence of elevated thyroid peroxidase or postoperative histopathology, collectively accrued as thyroiditis. A high normal preoperative TSH (HN-TSH) was defined as ≥2.01 uIU/ml, and a low normal TSH (LN-TSH) was defined as <2.01 uIU/ml. Class II/III obesity was defined as a Body Mass Index (BMI) ≥35kg/m2.

Thyroid function tests were obtained on all patients before operation to confirm a euthyroid state. Postoperative hypothyroidism was defined as TSH> 4.5 uIU/mL, or the use of thyroid hormone supplementation derived from medical records or telephone interviews. Time to hypothyroidism was determined by elevated TSH of more than 4.5 uIU/ml and medical records documenting the time of initiating thyroid hormone supplementation, early (<3 months) or late (> 3 months after operation).

Hypertension was defined as taking medication for antihypertensive medications; diabetes was defined as taking medication for glycemic agents; hypercholesterolemia was documented if patients were started on medication or had a prior diagnosis made in their medical record; and obesity/ BMI was calculated based on weight in kilograms (kg) and height in meters squared (m2) at time of surgery.

Statistical analysis

Statistical software STATA version 11.1 (StataCorp, College Station, USA) was used for the anlaysis [6]. A p-value of <0.05 was considered statistically significant. The independent effect of pre-operative TSH, BMI, thyroiditis, age at surgery, and other co-morbid diagnoses such as hypertension, diabetes, and hyperlipidemia was analyzed using bivariate analysis and multivariate analysis to adjust for the relative effect of these individual variables on the risk of post-operative hypothyroidism. Kaplan-Meier survival analysis was used to estimate and compare hypothyroidism-free survival at the bivariate level. Cox proportional hazards regression analysis was used to identify and adjust for predictors for the risk of hypothyroidism over time.

## Results

The characteristics of the study population are shown in Table 1.

**Table 1:** Demographics, co-morbidities, and rates of hypothyroidism after bivariate analysis *statistically significant, HN-TSH (high-normal thyroid stimulating hormone), HTN (hypertension), DM (diabetes mellitus), BMI (body mass index, kg/m2)

	Patients	Patients Hypothyroid (%)	P value
*HN-TSH			
No	134(78.8%)	36 (26.9%)	
Yes	36 (21.2%)	27 (75.0%)	<0.001
Total thyroiditis			
No	142(83.5%)	49(34.5%)	
Yes	28(16.5%)	14(50.0%)	P=0.121
*Age			
<45	70(41.2%)	17(24.3%)	
≥45	100(58.8%)	46 (46.0%)	0.004
Gender			
Male	30 (17.6%)	8(26.7%)	
Female	140 (82.3%)	55(39.3%)	0.194
*BMI			
<35	142(83.5%)	47(33.1%)	
≥35	26 (15.3%)	14(53.9%)	0.043
DM			
No	157(92.9%)	56(35.7%)	0.321
Yes	12(7.1%)	6(50.0%)	
HTN			
No	132(78.1%)	44(33.3%)	0.088
Yes	37(21.9%)	18(48.7%)	
Hyperlipidemia			
No	124(73.8%)	41(33.1%)	0.083
Yes	44(26.2%)	21(47.7%)	

Over the almost eight-year period, 170 hemithyroidectomies were performed for benign disease on patients with no prior thyroid insufficency. About 82% (140/170) were women, and the average age of males was significantly higher than females (55.1 vs 45.2 years, p<0.001). The majority of the cohort (58%) was >45 years old. The average age at operation was 47.0 years with SD ±14.1 years. All patients were euthyroid prior to surgery (pre-operative TSH values mean 1.39 uIU/ml, SD ±​​​​​​​0.85, Range 0.16 - 4.0). The mean follow-up was 22.7 months and the median was 13 months. Overall, 15.5% had a BMI of ≥35 and 36% of patients had an HN-TSH prior to the operation (Table 1).

**Figure 1:**
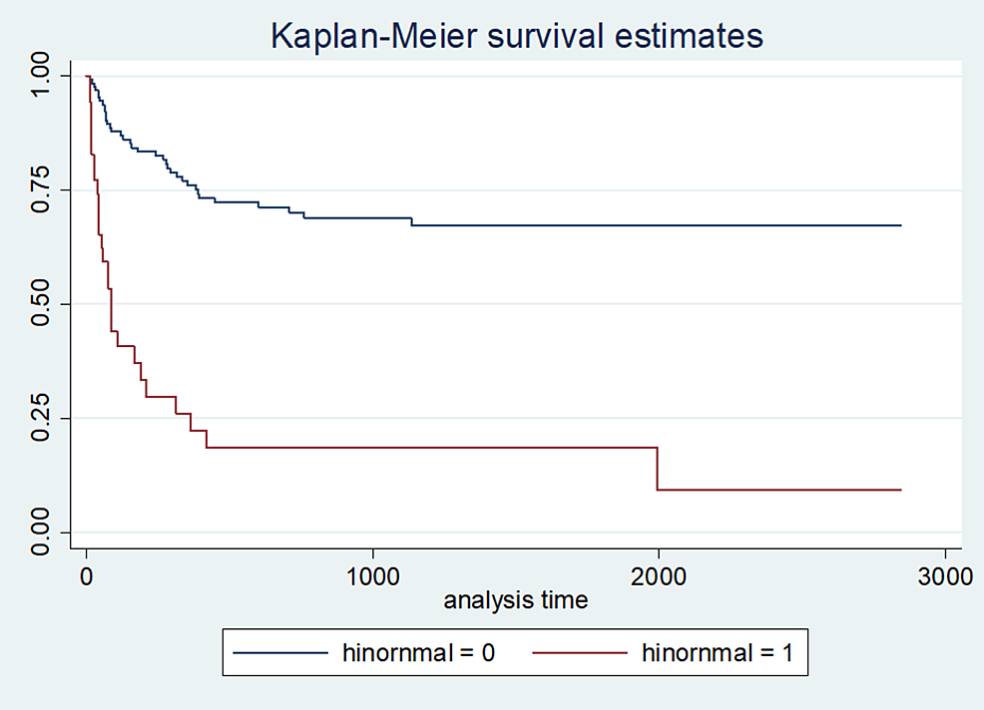
Kaplan-Meier curve for time to hypothyroidism in patients with HN-TSH Survival = proportion remaining euthyroid at the end of the study HN-TSH (high normal thyroid stimulating hormone)

Overall, 63 patients (37.1%) became hypothyroid during the eight-year study period. The overall hypothyroid-free survival (HFS), defined as the cumulative proportion of euthyroid patients at the end of the study was 52.4%; while the 1-year, 3-year and 5-year cumulative proportion of euthyroid patients were 65.7%, 58.5%, and 57.1%, respectively (Figure 1).

On bivariate analysis, age >45, BMI >35, and HN-TSH were significantly associated with the early development of hypothyroidism (the first 3 months) (p<0.05) (Tables 1-4). 

At 3 months, 21.5% of patients were hypothyroid. The unadjusted predictors of early progression to hypothyroidism within the first 3 months after the operation were age at surgery ≥45, TSH ≥2.01 uIU/ml (HN-TSH), and BMI ≥35 (p<0.05).

**Figure 2:**
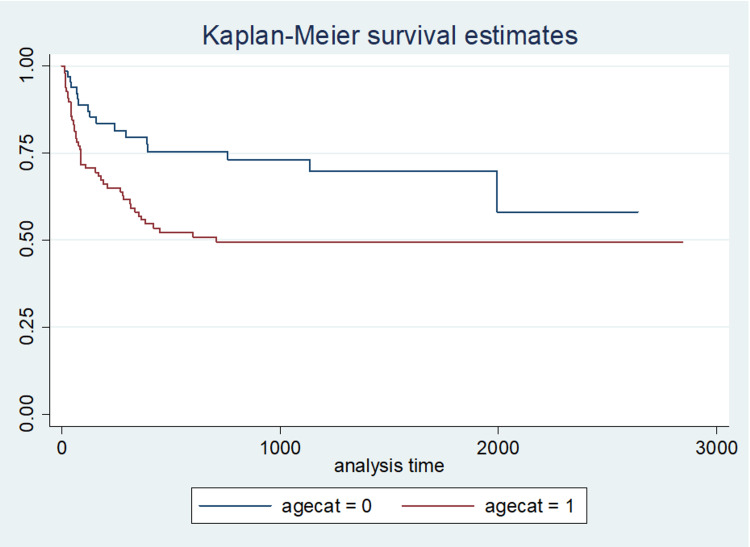
Kaplan-Meier curve for time to hypothyroidism in patients >45 years old

**Figure 3:**
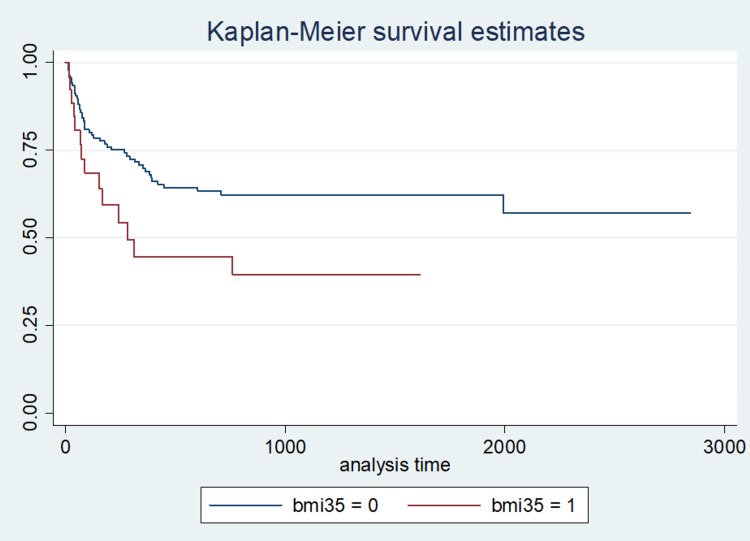
Kaplan-Meier curve for time to hypothyroidism in patients with BMI >35

After 6 months, 26.4% of the cohort was hypothyroid. And after 1 year 34.3% was hypothyroid. At 1 year the cumulative proportion of patients with hypothyroidism was 30.2% for BMI <35, 55.5% for BMI ≥35, 20.4% for age < 45, 43.1% for age ≥45, 26.9% for LN-TSH and 75% for HN-TSH (Figures 2-3).

On multivariable adjustment for the entire study period, the only independent predictor of overall conversion to hypothyroidism were HN-TSH (Hazard Ratio: 5.7, 95% confidence interval: 3.4 to 9.5, p<0.001) and hypertension (Hazard Ratio: 1.9, 95% confidence interval: 1.1 to 3.4, p=0.019).

## Discussion

The diagnosis of thyroid nodules and thyroid cancer is also increasing, and in 2013 the number of thyroidectomies performed annually in the United States was 31% greater than 5 years earlier [7].

The indications for hemithyroidectomy include compressive symptoms secondary to unilateral gland enlargement and “indeterminate” pathology on fine needle aspiration (FNA) [8]. The benefit of hemithyroidectomy is the preservation of functioning thyroid tissue, minimizing the risks of recurrent laryngeal nerve injury and hypoparathyroidism [9]. The disadvantages of a hemithyroidectomy include the potential need for a second operation if malignancy is found, and the lack of a guaranteed postoperative euthyroid state. Previously identified predictors of hypothyroidism after hemithyroidectomy have included a high normal pre-operative TSH level, a low pre-operative T4 level, the presence of Hashimoto’s lymphocytic thyroiditis, and small remnant thyroid volume [10].

Hypothyroidism may not appear immediately, and the diagnosis is discovered in some patients at 6-12 months after the operation. Previous studies have not included long-term follow-ups of these patients to determine the incidence of delayed hypothyroidism.

In our study, 63 of the 170 patients (37%) became hypothyroid during the eight-year study period, comparable to other studies. Miller et al identified a rate of 27% (24/90 patients), and more than 75% of their cases developed within the first 9 months [11]. McHenry and Slusarczyk identified a rate of 35% (25/71 patients) [12]. Stoll et al showed a rate of hypothyroidism of 14.3% (78/547) at 6-8 weeks after surgery [5]. We attribute the higher incidence of hypothyroidism in our patients to the longer follow-up time. In our cohort, the mean time to hypothyroidism was 6.6 months.

Patients aged >45 years developed hypothyroidism late (>3 months) when compared to the younger patients aged <45 years (p=0.01). Patients with a higher BMI (>35) developed hypothyroidism within the first 3 months of operation, compared to those with a BMI <35 (p=0.031). At 3 months, 21.5% of the patients were hypothyroid. The unadjusted predictors of early hypothyroidism were age at surgery ≥45 years, TSH ≥2.01 uIU/ml (HN-TSH), and BMI ≥35 kg/m2 (p<0.05). At 6 months after operation, an additional 5% had become hypothyroid, and after 1 year, 8% more were hypothyroid. Hypothyroidism occurred an average of 7.0 months after operation. The only independent predictor of hypothyroidism was preoperative HN-TSH (p<0.001).

Given the retrospective nature of this study, the presence of thyroiditis, which was mainly identified on pathology, could not be used as a predictor of hypothyroidism as this information was made known to us post-operatively. Blood work for autoimmune markers was not checked regularly for this study.

In our study, 37% of patients became hypothyroid after hemithyroidectomy, which is comparable to other studies [12,13]. The advantage of our study is that patients were identified close to the exact time point that thyroid hormone replacement was initiated. The majority of patients were also followed for over 2 years.

Age and BMI seemed to have an independent effect on predicting hypothyroidism. The importance of proper statistical methods is noted because after adjusting for HN-TSH, the effect of age and BMI were no longer significant. It is likely that age and BMI exert their effects through HN-TSH.

There is an increased incidence of HN-TSH among patients >45 years old, an interesting trend. Among patients aged <45, those with HN-TSH comprised 10% of the cohort, and those aged >45 years with HN-TSH made up 29% (p=0.003).

It is known that in older patients, there is an increased incidence of subclinical hypothyroidism, with mildly elevated TSH [14]. 25% of the cohort that developed early onset hypothyroidism were age ≥45 years (CI 0.78 to 0.95). However, age was not an independent predictor, which is consistent with McHenry et al. who also did not identify age as a predictor of hypothyroidism after hemithyroidectomy [12].

This paper has a follow-up of over 8 years, which is unique to this study. Our findings support that a delayed presentation of hypothyroidism is unlikely after the first year of operation, even for patients >45 years old. The presence of diabetes or hyperlipidemia was not significantly associated with greater hypothyroidism post-hemithyroidectomy.

Class II/III obesity was present in 15.3% of patients (26/170). Among these, 14/26 (53.9%) became hypothyroid post-hemithyroidectomy on bivariate analysis (p=0.04). To our knowledge, this study is the first study to demonstrate that obesity Class II/III (BMI ≥35) is associated with post-hemithyroidectomy hypothyroidism incidence on the bivariate. These predictors of hypothyroidism (BMI >35, age >45, and HN-TSH) should be incorporated into the discussion of surgical risk involved with hemithyroidectomy.

Our study is not without limitations. It is limited by its retrospective aspect and the low number of patients in the sample presented.

## Conclusions

Patients with age >45 years, BMI >35 kg/m2, and HN-TSH are at increased risk for early hypothyroidism after hemithyroidectomy and should be counseled on this increased risk. Knowing the time to hypothyroidism can help predict which patients need closer follow-up in the postoperative period and beyond. In the first year after hemithyroidectomy, 34.5% of patients became hypothyroid as the rate of hypothyroidism continues to be high up to 1 year after surgery. Therefore we recommend these patients should be followed up with routine thyroid function tests up to a year after hemithyroidectomy and later on as indicated by their symptoms.
